# The Expanding Universe of Extensions and Tails: Ribosomal Proteins and Histones in RNA and DNA Complex Signaling and Dynamics

**DOI:** 10.3390/genes16010045

**Published:** 2025-01-01

**Authors:** Youri Timsit

**Affiliations:** 1 Aix Marseille Université, Université de Toulon, CNRS, IRD, MIO UM110, 13288 Marseille, France; youri.timsit@mio.osupytheas.fr; 2 Research Federation for the Study of Global Ocean Systems Ecology and Evolution, FR2022/Tara GOSEE, Rue Michel-Ange, 75016 Paris, France

**Keywords:** ribosome, chromatin, network, signaling, dynamics, intrisically disordered protein

## Abstract

This short review bridges two biological fields: ribosomes and nucleosomes—two nucleoprotein assemblies that, along with many viruses, share proteins featuring long filamentous segments at their N- or C-termini. A central hypothesis is that these extensions and tails perform analogous functions in both systems. The evolution of these structures appears closely tied to the emergence of regulatory networks and signaling pathways, facilitating increasingly complex roles for ribosomes and nucleosome alike. This review begins by summarizing the structures and functions of ribosomes and nucleosomes, followed by a detailed comparison highlighting their similarities and differences, particularly in light of recent findings on the roles of ribosomal proteins in signaling and ribosome dynamics. The analysis seeks to uncover whether these systems operate based on shared principles and mechanisms. The nucleosome–ribosome analogy may offer valuable insights into unresolved questions in both fields. For instance, new structural insights from ribosomes might shed light on potential motifs formed by histone tails. From an evolutionary perspective, this study revisits the origins of signaling and regulation in ancient nucleoprotein assemblies, suggesting that tails and extensions may represent remnants of the earliest network systems governing signaling and dynamic control.

## 1. Introduction

The aim of this short review is to connect two seemingly distinct biological fields that are typically studied in isolation: ribosomes and nucleosomes [1,2]. These two nucleoprotein assemblies, along with many viruses, share a notable feature—the proteins that constitute them possess long filamentous segments at their N- or C-termini. These segments, often undergoing disorder-to-order transitions, are referred to as “extensions” in ribosomal proteins (r-proteins) [3,4,5] and “tails” in histones [6], which compose the chromatin filament. Intriguingly, the use of two different terms to describe a similar structural feature—a filamentous protein segment—highlights the linguistic and conceptual divide between these fields. This divergence, akin to Darwin’s finches adapting to distinct environments, suggests that ribosome and chromatin researchers have largely operated in separate domains, with limited cross-disciplinary communication.

Despite the differing nomenclature, histone tails and ribosomal extensions share numerous mechanistic, structural, functional, and evolutionary characteristics. Ribosomes and nucleosomes are central hubs of cellular and organismal regulation [7,8]. Their composition, compaction, and Post-Translational Modification (PTM) states define either the recently recognized ribosome heterogeneity [9,10] or the degree of chromatin condensation [11]. These states, which vary in response to different contexts, regulate processes such as specific gene expression and cellular metabolism, as well as organismal differentiation and epigenetics [12]. It is likely that the similarities between tails and extensions reflect shared mechanisms rooted in the interplay between their dynamic properties (disorder-to-order transitions) and nucleic acid compaction. In both systems, the protein filaments undergo disorder-to-order transitions that are intricately tied to the folding of nucleic acids—rRNA in the ribosome and DNA in compacted nucleosomes. These tails or extensions adopt well-defined conformations, folding into specific shapes that fit precisely within the crevices or cavities formed by the compacted RNA or DNA helices. Ultimately, their folded forms create distinct three-dimensional amino acid motifs with precise electrostatic properties, which may play a pivotal role in the functional architecture of the mature ribosome or the compacted nucleosomes. This parallel is particularly interesting in the light of recent studies that have revealed their roles in both controlling signaling and the dynamics of nucleoprotein interactions [13,14] in chromatin and in the ribosome [15,16,17].

The evolution of tails and extensions appears closely linked to the development of regulatory networks and signaling pathways, enabling increasingly complex functions for both ribosomes and chromatin. For example, the sophisticated extension networks in eukaryotic ribosomes or mitochondrial ribosomes [18,19,20,21] correspond to their expanded roles in these systems. Similarly, histone tails have evolved to regulate complex genetic systems, particularly in the transition from unicellular to multicellular life and the emergence of differentiation [22]. This parallel highlights a fascinating connection between the principles of nucleic acid compaction in the two systems and the regulatory roles of extensions and tails in their dynamics and signaling. A detailed comparative analysis of these similarities and differences could help uncover whether they share common origins or mechanisms, especially in the contexts of allostery and signaling [23,24].

Despite their structural and mechanistic parallels, the functional outcomes of these systems are fundamentally opposed. In ribosomes, the mature and most condensed forms facilitate protein synthesis, whereas in chromatin, more compact states are associated with the repression of gene expression. Remarkably, these opposing functions are both governed by the interplay between nucleic acid compaction and the filamentous protein segments that interact with them. This suggests that while extensions and tails may assist in nucleic acid condensation through similar mechanisms, their ultimate functional effects are contrasting. Bridging the fields of nucleosomes and ribosome research may provide complementary insights and help resolve unanswered questions about their mechanisms and evolutionary trajectories. For instance, mature ribosomes showcase an extensive repertoire of folded and structured extensions involved in interactions with rRNA, other proteins, or other extensions. These interactions, now observable in high-resolution structures [25], often captured at various stages of ribosome dynamics or maturation [26,27], could provide valuable insights into the structural and dynamic properties of histone tails, which remain less well characterized. Examining how ribosomal extensions transition from intermediate assembly states to mature forms may shed light on the structuring of histone tails during chromatin compaction. This review will first summarize the structures and functions of ribosomes and chromatin, followed by a point-by-point comparison to elucidate their similarities and differences. This analysis aims to advance our understanding of whether these systems share underlying principles and mechanisms.

## 2. Ribosome Signaling and Dynamics: The Interplay of rRNA and r-Protein Extensions

### 2.1. Ribosomes as Windows to the Past

Ribosomes are ribonucleoprotein particles that carry out translation—the mRNA-coded synthesis of proteins—across all three domains of life [28,29,30,31]. Cytoplasmic ribosomes consist of two major subunits, the large subunit (LSU) and the small subunit (SSU) with a composition of approximately one-third protein and two-thirds RNA. The resolution of ribosomal structures from the three domains of life, including their various substrates and associated factors, has provided a wealth of data about the mechanisms and dynamics underpinning the four translation stages: initiation, elongation, termination, and recycling. Additionally, studies of ribosome biogenesis yielded valuable insights into the concerted assembly of nucleoprotein complexes, shedding light on mechanisms and evolutionary trajectories [32,33]. Over the past two decades, structural, biochemical, and theoretical studies revealed the intricate dynamics that ensure the high fidelity of protein biosynthesis [34,35,36,37,38,39]. Comparative studies of ribosome structures across the three domains demonstrated how ribosomal complexity has increased over evolutionary time. Often referred to as a “window to the past”, ribosomes offer an intriguing system for studying evolutionary transitions [40,41,42,43,44,45]. Some hypotheses propose that ribosomes represent autonomous systems that predate the divergence of the three domains and may serve as a missing evolutionary link [46,47]. According to these views, ribosomal structure reflects the “first steps” in the evolution of a peptide/RNA world, marking the transition from the RNA world to the first peptide/RNA life forms [48,49,50]. Ribosomal organization is thought to illustrate the co-evolution of translation machinery and the genetic code [51,52,53]. With their unique repertoire of rRNA modules [54,55,56,57] and ancestral proteins, ribosomes may represent the earliest forms of organization and mutualism between RNA and peptides [58]. As such, they likely lie at the crossroads of evolutionary processes predating the Last Universal Common Ancestor (LUCA) [59], marking the end of the so-called “RNA world”. Numerous studies suggested that ribosomes evolved through accretion, with their components following distinct evolutionary pathways before coalescing into modern ribosomes [40,60].

Ribosomal RNA (rRNA) and ribosomal proteins (r-proteins), the key constituents of the two subunits, appear to have different origins and co-evolved to form today’s ribosomes [41,61,62]. The evolution and phylogeny of r-proteins were explored extensively, revealing complex evolutionary scenarios across the three domains [63,64,65,66] and based on the gradual evolution of protein motifs [67,68,69]. R-proteins are particularly notable for their unique structure, comprising a globular domain that typically anchors to the ribosomal surface and long, disordered, filamentous extensions that weave through the rRNA crevices that are frequent structural features of large RNA assemblies [70,71]. The roles of these extensions that have been already noticed in the first high-resolution crystal structures of ribosome remain enigmatic [29]. Due to their charged and dynamic nature, these extensions are hypothesized to aid in ribosome assembly [3,4,5,72,73,74]. In addition to their interactions with rRNA, r-protein extensions systematically form inter-protein networks through tiny but well-conserved interactions within the ribosomes of all three domains [15,75]. Unlike transient protein networks commonly observed in cells [76,77], r-protein networks are characterized by permanent interactions [15] within mature ribosomes after biogenesis is complete [78,79,80].

**Figure 1:**
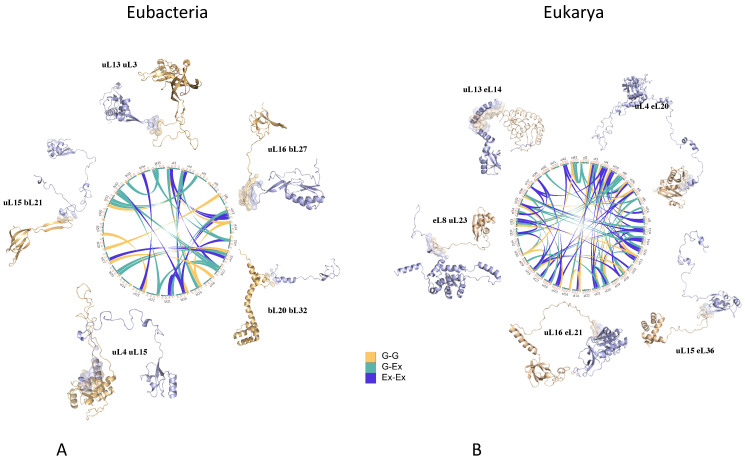
An inexorable expansion of the r-protein extension universe. Circos representation show that the r-protein extensions form network whose complexity increase form prokaryotes to eukaryotes. Network connectivity (**A**) in eubacterial ribosome and (**B**) in eukaryotic ribosome. Each circle is surrounded by structural examples of interactions of r-protein through their extensions depicted with cartoons. Color codes for connections in the circles: G-G: contact between r-protein globular domains (yellow); G-Ex: contact between r-protein globular domains and extensions (green): Ex-Ex: contact between r-protein extensions (blue).

The structural comparisons of ribosomes from the three domains (archaea, bacteria, and eukaryotes) has shown how these network have evolved through the gradual acquisition of r-protein extensions [16] (Figure 1). This study also inferred the existence of a universal r-protein network that likely existed in LUCA [81] before the kingdoms diverged. This ancestral network appears to have been functionally connected to key sites such as the Peptidyl Transferase Center (PTC), mRNA, tRNAs, and the peptide exit tunnel. Over time, this network expanded, reaching its highest connectivity in eukaryotic ribosomes. Each domain’s network has followed distinct evolutionary pathways, resulting in variations in complexity but retaining a common architecture shaped by divergent and convergent evolution [16].

### 2.2. Ancient Signaling Systems at the Heart of the Ribosome

Experimental evidence increasingly supports the idea that distant ribosomal functional sites continuously sense and transmit molecular signals. For example, long-range signaling between the decoding center ensures proper codon-anticodon geometry while interacting with distant regions such as the Sarcin-Ricin Loop (SRL) or the E-tRNA site [82,83]. For example, r-proteins such as uL3 play critical roles in coordinating the PTC [84] and A-site [85], while r-proteins that sense nascent peptides within the exit tunnel regulate co-translational folding and communicate with remote sites like the PTC [86,87]. These communication processes also synchronize complex ribosomal movements during translation, including the ratchet-like motions of the two subunits [88,89,90]. Although ribosomes are considered ribozymes [91], and r-proteins have traditionally been viewed as auxiliary components primarily involved in biogenesis [78,92,93,94], several studies indicate that they may combine several functions. Mutagenesis studies suggest that specific extensions, such as those in uS12, eL8, uL29, and uL30, play crucial roles in both assembly and translation [74,95,96,97,98,99]. Numerous studies, including those by Dinman’s group, demonstrate the importance of r-protein extensions in mediating long-range communication between functional sites [85,100,101,102,103,104,105,106], coordinating efficient translation, and linking to signaling pathways [107,108]. The dramatic expansion of r-protein networks across the tree of life provides a framework for integrating evolutionary, structural, and functional data (Figure 1).

Studies suggested that r-proteins evolved collectively, gradually adopting roles in transmitting and processing signals between distant ribosomal sites during translation. Similarly, rRNA also participates in long-range communication [109,110,111,112], co-evolving with r-proteins to facilitate information exchange [15,16,75]. This expansion in r-protein network connectivity parallels the increasing accuracy and complexity of ribosomal tasks from prokaryotes to eukaryotes [113,114,115]. Alterations in network connectivity are likely to impact translation efficiency and accuracy, offering insights into ribosome heterogeneity and its regulation of translation, as well as its links to diseases [9,10,116].

**Figure 2:**
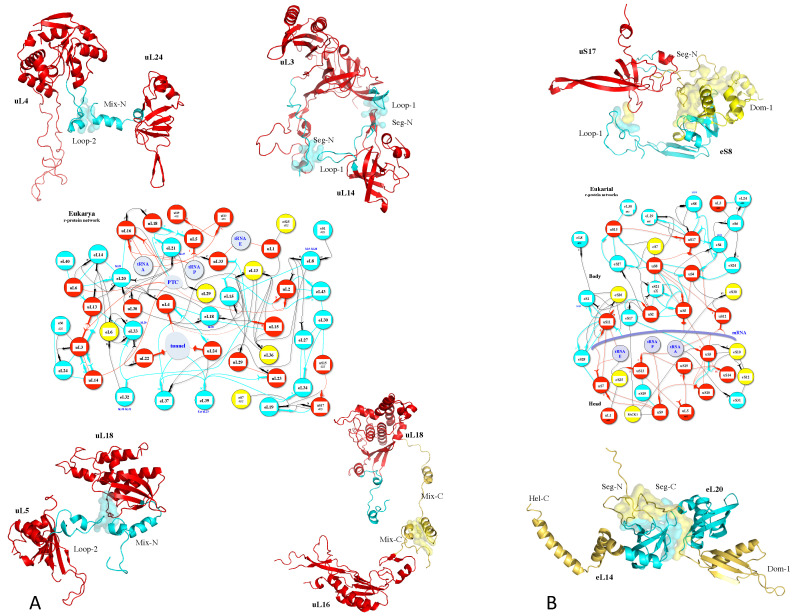
Co-evolution of extensions for networking in the eukaryotic ribosome. (**A**) LSU; (**B**) SSU. The proteins and their extensions are colored according to their evolutionary status. Red: universal (observed in bacteria, archaea and eukarya); Cyan: archaea (r-protein or extensions acquired in the archaeal ribosome and maintained in the eukaryotic ribosome); Yellow: eukaryote (r-protein or extension acquired in eukaryotic ribosome. The figure shows r-proteins that have co-acquired new extensions for networking at a defined evolutionary transition. For example, the universal r-proteins uL4 and uL24 (**top left**) have both acquired new extensions in the archaeal ribosome (cyan) that interact together.

A striking finding is that ribosomal extensions evolved concurrently across distant r-proteins to establish new connections [15,16] (Figure 2). This coordinated evolution, termed co-evolution [117,118], was significantly amplified during the transition from archaea to eukaryotes. It was observed that in general, the acquisition of new protein insertions correlates with network rewiring [119,120,121]. At the amino acid level, the emergence of motifs indicative of r-protein contacts aligns with the appearance of novel inter-protein interactions. For example, in *Mycobacterium smegmatis* and *Escherichia coli* ribosomes, new motifs are located near interaction points of recently acquired extensions [25,122]. Interestingly, while extension-mediated protein-protein interactions increased significantly from prokaryotes to eukaryotes, we noticed that extension-rRNA interactions exhibited only modest growth, thus further demonstrating that r-protein extensions have co-evolved for networking [16]. In eukaryotes, kingdom-specific extensions predominantly interact with other proteins rather than with eukaryote-specific RNA expansion segments.

**Figure 3:**
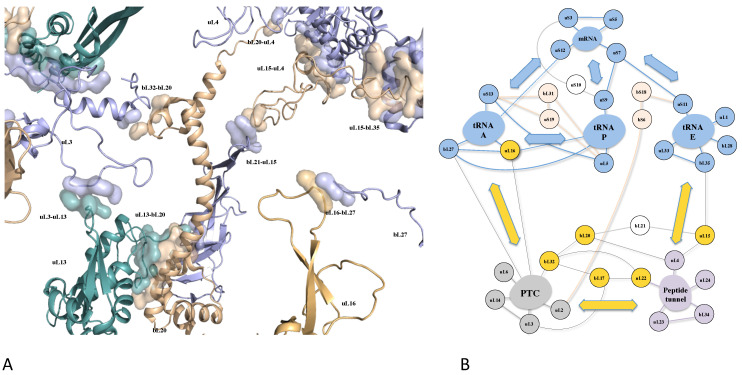
(**A**) view of the r-protein network of the large subunit of the bacterial ribosome (pdb_id: 4v9f). The rRNA is not represented to highlight the tiny protein interactions. (**B**) Schematic representation of connectivity between the functional modules, emphasizing that the network has evolved to interconnect the functional centers of the ribosomes.

This suggests that the primary evolutionary role of extensions lies in enhancing protein-protein interactions. These phenomena, expressed at both protein and amino acid levels, highlight the strong selective pressures shaping r-protein networks for the allosteric transmission of various signals between ribosomal functional sites (Figure 3).

**Figure 4:**
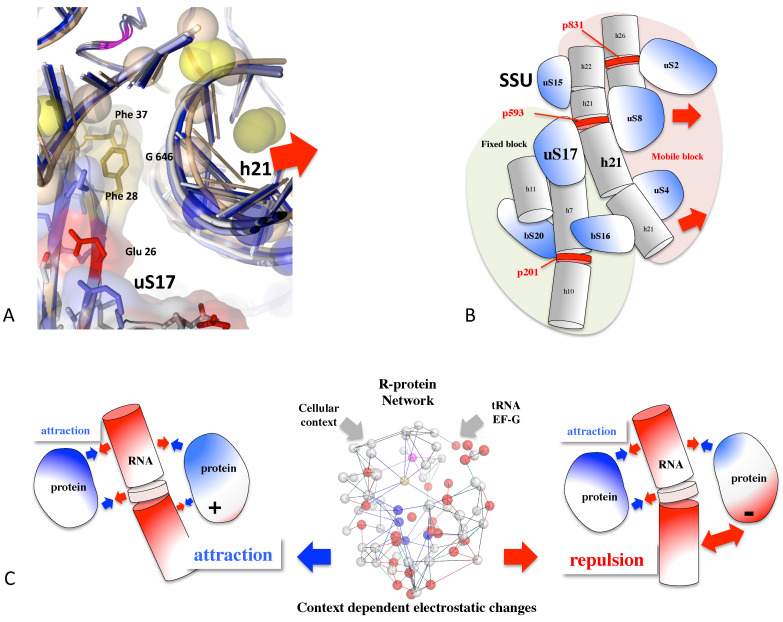
The roles of the r-proteins in the control of the bacterial ribosome dynamics during translocation. (**A**) The RNA helix h21 moves relatively to the r-protein uS17 in the small subunit (pdb_id: 7n1p, 7n30, 7n2u, 7n2v, 7n2c, 7n31). The blue spheres correspond to the moving phosphate groups (>1Å) during the translocation steps [17,26]. The side chains of the uS17 amino acids facing the moving rRNA are represented by sticks. (**B**) Schematic representation of the overall ribosome dynamic controlled by the uS17/h21 mobility. The red disks represent the base pairs that serve as joints around which the RNA segments are mobile. (**C**) An electrostatic model for the periodic protein/RNA distance approach cycle during the translocation. The surface potentials of r-proteins can fluctuate depending on context-specific changes transmitted through the r-protein network. Ionizable groups (His, Glu, Asp) can shift from being attractive to repulsive toward RNA.

A recent study demonstrated that the r-proteins also cooperate to control the overall ribosome dynamics during translocation. It was proposed that distance/approach cycles between rRNA and r-proteins are modulated by periodic electrostatic changes transmitted among r-proteins through their network. These coordinated changes may therefore adapt the ribosome dynamic to contextual events (Figure 4).

## 3. Histone Tails as Key Player in Chromatin Dynamic

### 3.1. Histones and the Structure of the Eukaryotic Nucleosome

Eukaryotic nuclear DNA is packaged into chromatin [1,11], where nucleosomes represent the fundamental units of this organization [123]. Each nucleosome comprises a histone octamer formed by two copies of the core histones H2A, H2B, H3, and H4, around which 147 base pairs of DNA wrap in approximately two superhelical turns [123,124,125]. This structure is highly conserved and crucial for genome stability, transcriptional regulation, DNA repair, and replication. The histones feature a characteristic “histone fold” motif, composed of three α-helices linked by two loops. This fold is essential for dimerization and the formation of the octamer through a “handshake” interaction. Histones possess flexible N- and C-terminal extensions, or “tails”, which protrude from the nucleosome core [6]. These tails, rich in positively charged residues, interact dynamically with DNA and neighboring nucleosomes, stabilizing chromatin architecture [126,127,128,129,130]. Despite their disordered nature, these tails play critical roles in chromatin dynamics by modulating inter- and intranucleosomal interactions, contributing to DNA accessibility, and transferring histone octamers along the DNA. The tails are also major sites of Post-Translational Modifications (PTMs) [12], which influence chromatin structure and function by altering histone properties or recruiting regulatory proteins.

The compaction levels and supramolecular structures of chromatin which are controlled by linker histones, DNA torsional stress and salt concentrations [131,132,133,134,135,136,137,138,139,140,141] significantly impacts DNA accessibility to the transcription machinery. These various states are thought to constitute a hub for genomic functions [142,143]. Histone tails mediate this process through dynamic interactions with DNA and other histones [14,126]. They participate in nucleosome-nucleosome contacts, aiding chromatin fiber compaction and oligomerization [127,130,136,144,145,146]. Among the core histones, the H4 tail has the most pronounced effect on chromatin condensation, followed by H3, while H2A and H2B tails play secondary but cooperative roles. These interactions not only neutralize DNA’s negative charge but also create specific inter-nucleosomal contacts crucial for chromatin stability [147,148]. For example, conformational changes in the H3 tail have been linked to chromatin condensation states and nucleosome composition [130,149,150].

### 3.2. Histone Variants, PTMs and the Histone Code

Post-Translational Modifications (PTMs) of histone tails are central to the regulation of chromatin dynamics and epigenetic control [12]. These modifications, which include acetylation, methylation, phosphorylation, and serotonylation, alter chromatin structure through direct (cis) effects on histone properties or indirect (trans) recruitment of regulatory proteins [13,151]. For instance, lysine acetylation neutralizes histone tails’ positive charge, weakening their interaction with DNA and promoting chromatin decompaction, which is conducive to gene activation. Methylation, depending on the specific residue modified, can either activate or repress transcription. The “histone code hypothesis” posits that specific PTM combinations create a molecular language decoded by enzymes known as “writers”, “readers”, and “erasers” [152]. These modifications orchestrate key processes, including transcription, DNA repair, and cell differentiation. Moreover, histone tails contribute to chromatin compaction through direct inter-nucleosome interactions. The H4 tail is particularly significant in mediating contacts between adjacent nucleosomes, enabling chromatin to adopt higher-order structures.

Histone variants, such as H2A.Z and H3.3, introduce additional layers of chromatin specialization by altering nucleosome dynamics. H2A.Z enhances DNA accessibility and transcriptional activity, while H3.3 plays a role in maintaining nucleosome stability [144,153]. The distinct tails of these variants influence their interaction with DNA and chromatin-associated proteins, conferring unique functions in processes like stress response, DNA repair, and regulation of gene expression.

### 3.3. Evolutionary Origins of Histones and the Histone Fold

Eukaryotic histones share a common evolutionary origin with archaeal histones, characterized by strong structural and sequence similarities [154]. In archaea, histones form dimers that wrap DNA into hypernucleosomes, which facilitate genome compaction and transcription regulation [155]. Some archaeal histones possess lysine-rich tails analogous to eukaryotic histone tails, suggesting that the regulatory potential of histones emerged before the divergence of these lineages. Recent studies indicate that histones and their PTM systems may date back to LUCA. For example, bacterial histones with fused functional domains, such as zinc-binding sites, highlight the evolutionary plasticity of these proteins [156]. The discovery of histones in bacteria and viruses further supports the hypothesis of an ancient origin predating the radiation of archaea, bacteria, and eukaryotes [67,157,158,159,160]. In eukaryotes, the emergence of the H2A-H2B heterodimer likely disrupted the formation of archaeal-like hypernucleosomes, leading to the evolution of chromatin as we know it today. The distinct structural features of H2A and H2B, such as the absence of dimer-dimer interfaces and the presence of additional helices, prevented hypernucleosome stacking and facilitated the dynamic regulation of chromatin organization. The evolution of histone tails reflects a progressive adaptation to increasing regulatory complexity in eukaryotes. Archaeal histones with lysine-rich tails may represent a transitional form, enabling more flexible chromatin interactions. For example, tails from archaeal phyla such as *Candidatus Heimdallarchaeota* exhibit lysine-rich regions, which may interact with DNA and proteins in ways analogous to eukaryotic histone tails [154]. While experimental evidence remains limited, these findings suggest a shared evolutionary trajectory between archaeal and eukaryotic chromatin regulation systems.

In summary, histones and their tails are central to chromatin organization, dynamics, and regulation. From their evolutionary origins in archaea to their sophisticated roles in eukaryotic chromatin signaling, histones exemplify how structural adaptations can drive functional diversity. Understanding their evolution and mechanisms continues to provide critical insights into genome regulation across the tree of life.

## 4. Aligning Functions of Extensions and Tails

### 4.1. Compaction and Signaling in the RNA and DNA Worlds

Beyond the universality of the genetic code, the mode of compaction of nucleic acids is another fundamental feature of living organisms that is rarely thoroughly examined. This compaction not only allows for the condensation, protection, and regulation of genetic information but also plays a crucial role in how this information is stored and controlled. While the base-pairing complementarity has been a determining factor in the choice of nucleic acids as genetic information carriers [51,161,162], the mode of compactions of the different forms of RNA or DNA double helices has also influenced the organization, the regulation of these structures and their recognition by proteins [163,164,165,166,167]. The primary constraint related to base pairing established the fundamental principles of replication and, consequently, the genetic code. In an another hand, chirality, geometry, and electrostatic properties of nucleic acid double helices had major consequences on the supramolecular architecture of their condensed forms and on their regulation by proteins since the origins of life [164]. These structural characteristics not only affect the tertiary organization of genetic information but also how protein interactions have evolved to modulate compacted structures.

The chirality of nucleic acid helices, resulting from the double-helix configuration, imposes packing rules that constraint the geometry of their supramolecular assemblies. These rules have implications for the solutions adopted by evolution to compact nucleic acids, moving from ancient forms based on RNA, such as the ribosome to modern one such as the chromatin based on DNA. Thus, the transition from the RNA world, capable of self-replication and catalyzing certain biochemical reactions, to the DNA world is linked to the distinct structural properties of ribose (RNA) and deoxyribose (DNA). Paradoxically, due to the ability to form non-canonical base pairs and complex tertiary interactions [55,56,168], the RNA assemblies are much more complicated and diverse than compacted DNA forms that display a great sobriety [164,167,169]. This structural difference, particularly the presence or absence of the 2-OH group on ribose, has profound consequences for the stability and compaction properties of nucleic acids. A-form helices, found in RNA, and the B-form of DNA, which is the dominant form in modern cells, differ in their condensation and packing rules. The invention of DNA allowed for the uniformity of the double helix geometry, creating simpler structures with interactions between helices dominated by the electrostatic repulsion of the sugar-phosphate backbone. These interactions vary depending on whether the helices align in parallel columns or cross over to adjust their structures within the grooves. The simplification of packing rules allowed the organization of large genomes of modern life forms. Without compaction, chromosomal DNA would occupy a volume up to 1000 times larger than that of the cell, underscoring the necessity of chromatin organization and condensation.

Despite their differences, RNA and DNA compaction share common principles in both the ribosome and chromatin. To be functional, these nucleoprotein complexes must achieve a balance between stability and the ability to modulate their dynamics. Compacted forms of nucleic acids are characterized by unique geometries and electrostatic properties, creating highly negatively charged cavities and grooves stabilized by cations. These features may have influenced the flexible and positively charged nature of r-protein extensions. The compact forms of nucleic acids could have first served as molds for shaping the first peptide sequences during the early stages of the RNA world, leading to the emergence of peptide/RNA complexes. While one of the essential roles of these peptides is to stabilize the compact forms of nucleic acids, an equally important function is to control the dynamics of these assemblies. It is therefore possible that the sequences of these flexible structures have also been evolutionarily optimized to enable the regulation of their interactions with RNA and the dynamic of nucleoprotein complexes.

### 4.2. Chromatin and Ribosome Dynamics: Variations on a Common Theme

Nucleosome and chromatin are indeed now recognized as dynamic and multifunctional entities. As seen above, experimental and theoretical studies indicate that histone tails and their PTM play a crucial role in the dynamics and stability of nucleosomes, as well as chromatin condensation [126,127,128,170,171]. PTMs at specific sites on extensions and DNA (e.g., DNA methylation and modifications of histone tails) play a critical role in modulating how tails influence chromatin structure and dynamics [172,173,174,175,176]. Similarly, the rRNA undergo various steps of compaction during its biogenesis displaying various intermediate states [177,178,179,180] where r-proteins extensions and rRNA modifications play a critical role [181,182].

Comparing these studies highlights similarities between chromatin compaction and ribosomal assembly intermediates, underscoring shared fundamental principles between these two structures. Particularly interesting are the existence of similar mechanism of PTM and the use of “variants” in the composition of both chromatin and ribosomes, for controlling their compaction processes [22,152,183,184,185,186,187,188,189]. Thus, both histones and r-proteins exert a tunable electrostatic influence on the structure and dynamics of supramolecular assemblies. The evolution of epigenetic mechanisms or PTMs appears to be similar in ribosomal systems and chromatin.

### 4.3. Rudimentary Low-Complexity Segments or Allosteric Geniuses?

R-protein extensions and histone tails are characterized by their ability to adopt multiple structures that can be shaped according to specific contexts and in particular nucleic acid compaction. Often, they are considered Intrinsically Disordered Proteins (IDPs) because, in the absence of binding partners, they can adopt a variety of conformations or exist as a conformational ensemble [70,190,191,192]. However, when these extensions or tails interact with other proteins or nucleic acids, they can form well-defined structures. For example, the structures of ribosomes across the three kingdoms provide striking examples of how these extensions are folded into the cavities between rRNA helices and how they interact with other extensions, forming complex networks. As demonstrated in our recent study, the r-protein extensions combine properties that enable them to perform multifunctional roles [17]. While their flexibility can facilitate the ribosome assembly steps, the clusters of positive charges help neutralize the repulsion caused by the proximity of phosphate groups during rRNA compaction. As shown above, the r-protein extensions may also play an allosteric role, contributing to the transmission of signals between distant functional sites within the ribosome (Figure 1). However, we have also shown that r-protein allow for a context-dependent regulation of RNA/protein dynamics, and consequently, the dynamics of the ribosome during translocation. R-proteins were catalogued and classified, revealing an intriguing diversity of structural motifs, including a recurrent combination of aromatic and negatively charged amino acids. As highlighted in this recent research, the r-protein network plays a fascinating dual role in ribosome signaling and in regulating rRNA dynamics. R-proteins fulfill far more complex functions than merely stabilizing rRNA. First, highly conserved negatively charged residues (Glu and Asp) are frequently observed in close proximity to rRNA backbones. This suggests that these r-protein surfaces perform functions beyond simple electrostatic neutralization of the negatively charged phosphate groups. Second, we identified significant RNA/protein mobility around Glu and Asp residues embedded in complex motifs that include histidine and other aromatic amino acids. Given that histidine can easily switch between positive and neutral states, we inferred that these motifs likely modulate their interactions with nucleic acids through “context-dependent electrostatic changes” (Figure 4C).

**Figure 5:**
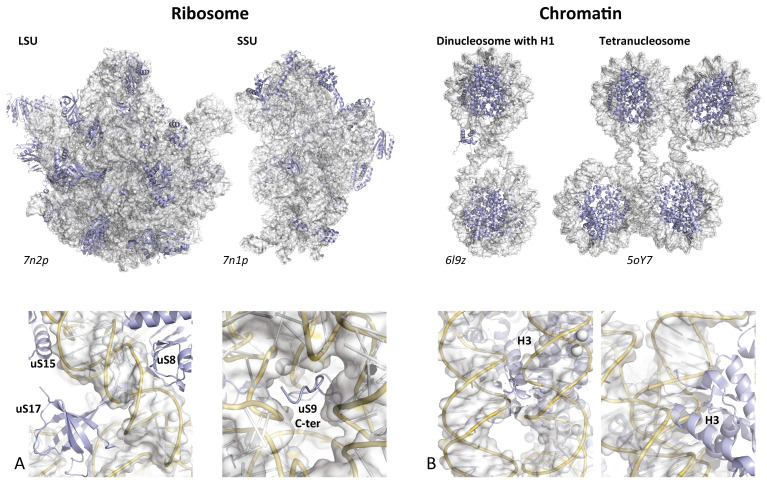
RNA and DNA supramolecular assemblies. (**A**) rRNA-rRNA (white surfaces) and r-protein-rRNA interactions within bacterial ribosomes. (**B**) DNA-DNA and DNA histones interactions with the various higher-order structures of chromatin. Bottom: details of the interactions showing the similar extensions (**A**) or tails (**B**) trajectories within the cavities formed between the compacted nucleic acids.

**Figure 6:**
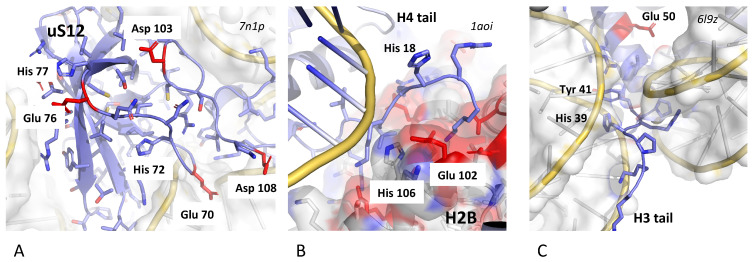
The role of ionizable groups (Glu, Asp and His) in the dynamics of ribosome and chromatin. Similar motifs containing negatively charged amino acids (Glu and Asp) and Histidine that can switch easily from positive to neutral are observed at the vicinity of nucleic acids in ribosomes (**A**) and compacted nucleosomes (**B**,**C**). Electrostatic fluctuations in these protein motifs may control the dynamic of ribosomes and chromatin.

The histone tails also fulfill within the cavities formed by DNA-DNA contacts in higher-order structures of chromatin (Figure 5 and Figure 6). Interestingly, in addition to their positively charged residues, they display motifs similar to that of the r-proteins, which may also modulate their interactions with DNA (Figure 6). For example, the first nucleosome structures offered structural insights into how the N-terminal tail of H4 may regulate nucleosome interactions and compaction by engaging with the nucleosome “acidic patch” [146,193,194] (Figure 6B). It was pointed in a recent review that histone tails may act collectively as “signaling antennas” and control the dynamics of higher order structures of the chromatin [13]. It is therefore likely that the common structural properties of r-protein extensions and histone tails reflect their multifunctional roles and evolutionary development.

Histone tails are now viewed as sets of metastable structures rather than strictly intrinsically unstructured proteins. Their energy landscapes suggest that the conformational landscape might be better understood as a collection of well-defined, competing, metastable structures, rather than complete disorder [195]. The same idea applies to the ribosome. The mature structures of the ribosome show snapshots of possible conformations that r-protein extensions can adopt. For instance, comparing an intermediate assembly state of the bacterial large subunit with its mature form shows that some extensions (i.e., uL3 and uL13) found in structured states in the mature form are disordered in the intermediate state.

Looking at the properties of extensions and tails in parallel could also extend the concept of IDPs and inspire a new way of considering disorder. It was suggested that the disorder in tails allows for the efficient capture of binding partners via the “fly-casting” mechanism [195]. Similarly, it has been proposed that the disorder in protein extensions might play a comparable role during ribosome assembly [72]). The disorder/order transitions observed in the bL20 protein, which is the first structure shown to exhibit both folded and unfolded states within the same crystal, illustrate how the flexibility of a protein segment can facilitate interaction with distant partners [73]. Disorder would be the (smart) key to multifunctionality that emerged at the origin of life, when the first nucleoprotein complexes already required a combination of stability, dynamic control, and signaling.

### 4.4. Evolutionary Aspects: Tails and Extensions as Windows to a Multifunctional Past

The relationship between histone tails and ribosomal protein extensions reveals intriguing evolutionary and functional parallels. Recent discoveries have challenged the old paradigms surrounding histone evolution. It has now been found that histone folds are present in several prokaryotic clades and probably emerged before the radiation of the three kingdoms [154,156,157,158]. These new findings draw them closer to r-proteins, which are emblematic representatives of the LUCA proteome. A possible conclusion of these new discoveries is that histones and chromatin originated with LUCA alongside r-proteins. This proposes a deep and shared origin for tails and extensions, reinforcing the hypothesis that they have a common functional origin for signaling and the dynamic control of nucleic acid assemblies. Thus, histones and chromatin inherited sophisticated signaling and movement control systems developed during the ribosome era. A interesting finding reinforcing this view is the similarity in sequence between the histone tail of mosquitoes and the extension of r-proteins [196,197,198]. This evolutionary convergence supports the idea that they may share similar roles. Histone H1 proteins in *Anopheles* mosquitoes exhibit significant sequence identity with the C-terminal extensions of ribosomal protein RPS6, suggesting potential functional overlap. Additionally, the presence of histone H1-like extensions in ribosomal proteins such as RpL23a and RpS6 indicates that these extensions could play roles in ribosomal assembly or function, even though they evolved independently.

## 5. Conclusions

Technically, the chromatin/ribosome analogy proposed in this review may provide insights into solving unresolved questions within each field. For instance, while histone tails are seldom observed in nucleosomes and compacted chromatin, r-protein extensions are well defined in the ribosomal structure. Information from the ribosome system can provide valuable clues regarding structural motifs that histone tails might form. This knowledge extends to a repertoire of elementary structural motifs created by individual extensions, the types of interactions they can engage in, and their network architecture. Such understanding could be applied to histone tail behavior and chromatin compaction. Conceptually, the recently uncovered roles and mechanisms of r-proteins in ribosome dynamics may also be applicable to chromatin dynamics. From an evolutionary perspective, the parallel between extensions and tails led us to the root of signaling and regulation of compaction. This short review suggests that tails and extensions are remnants of the earliest network systems responsible for signaling and the dynamic control of compacted nucleo-protein assemblies. These elements are variations on a common theme, the already well elaborated systems that may have existed at the origin of life for transferring, integrating and controlling motions [199,200]. Variations on a theme can generate a wide range of forms that display coherence and universality. This evolving process may converge towards an apparent simplicity. While modern genomes have simplified their packaging mode of DNA, they still conserve old and disordered tails or extensions that seem indispensable for controlling both signaling and the dynamics of nucleo-proteins interactions.
